# Person-centered, trauma-informed care and support for resilience after intimate partner violence: an integrative review through a neurobiological lens

**DOI:** 10.3389/fpubh.2026.1962315

**Published:** 2026-10-27

**Authors:** Nóra Kerekes, Caroline Ackemo, Erika Alfredsson, Anis Sfendla

**Affiliations:** 1 Institution for Health Sciences, University West, Trollhättan, Sweden; 2 Center for Holistic Psychiatry Research (CHoPy), Mölndal, Sweden; 3 High Institute of Nursing Professions and Health Techniques Fez, Dhar El Mehraz Hôpital Al Ghassani, Fez, Morocco

**Keywords:** care relationship, empowerment, gender inequality, resilience, social support, trauma neurobiology

## Abstract

**Background:**

Intimate partner violence (IPV) is a major global public health concern with severe physical, psychological, and social consequences, strongly associated with chronic stress and diminished wellbeing. IPV often remains under-identified within healthcare and social care settings, with shame, guilt, and stigma acting as barriers to help-seeking. Resilience—the capacity to regulate emotion, adapt, and recover following adversity—has emerged as a central concept in understanding recovery after IPV exposure. Person-centered, trauma-informed care facilities recovery by grounding care in the individual's needs, experiences, and resources and by accounting for how trauma affects the body and the capacity to trust care practitioners.

**Aim:**

The aim of this study was to analyze person-centered care and support interventions aimed at strengthening resilience in individuals exposed to IPV.

**Methods:**

An integrative literature review was conducted following Whittemdore and Knafl's methodology. It included 12 peer-reviewed studies published between 2015 and March 2026, retrieved from CINAHL and APA PsycInfo. Nine studies were qualitative, two quantitative, and one mixed methods; all appraised using the CASP framework.

**Results:**

Findings were based on 544 participants exposed to IPV (537 women, 7 men, aged 15–73 years), alongside the perspectives of 31 care and support professionals, drawn from studies conducted across eight countries. Three main themes were identified: a safe, trusting, and accessible care relationship as a foundation for resilience; empowerment through participation, autonomy, and reclaiming control; and the mobilization of individual and social resources for long-term recovery, encompassing strength-based coping and supportive networks. Person-centered, trauma-informed care characterized by safety, trust, and genuine participation was central to strengthening resilience. Organizational barriers, along with gendered dynamics affecting autonomy could hinder access to support and recovery.

**Conclusion:**

Person-centered, trauma-informed care and support can strengthen resilience by fostering trust, participation, and empowerment. Resilience emerged as a dynamic, nonlinear process shaped by individual resources, relationships with care and support staff, and structural conditions. Trauma's effects on the nervous system offer a neurobiological basis for why consistency, predictability, and genuine attentiveness are prerequisites for effective care, not merely good practice. These findings underscore the need for training in trauma-informed, person-centered approaches across all professions supporting individuals exposed to IPV.

## Background

The World Health Organization defines intimate partner violence (IPV) as any behavior within an intimate relationship that causes physical, psychological, or sexual harm to any person in the relationship (1). This was later refined by the WHO, which described IPV as a pattern of acts in which a partner or former partner exercises physical, sexual, psychological, or economic violence with the purpose of establishing and maintaining power and control over another individual (2). IPV research has largely investigated women as victims and men as perpetrators, although this framing has increasingly been challenged by studies addressing men as victims, beginning with early national survey work documenting female-to-male violence (3) and continuing in more recent research (4, 5). Estimates of IPV prevalence vary depending on gender, sexual orientation, sociocultural context, and socioeconomic factors, but it remains clear that IPV is a gendered problem, with gender understood as more than an individual characteristic and shaped by broader societal expectations of behavior (6). Globally, one in three women is estimated to have been subjected to physical and/or sexual violence by a partner at some point in her lifetime. This prevalence has remained largely unchanged over the past two decades, underlining that IPV is one of the most widespread forms of violence against women worldwide (2, 7).

IPV entails significant suffering and a serious violation of human rights, constituting a major public health and societal problem that affects the individual's freedom, health, and well-being (2, 8, 9). Exposure to IPV is strongly linked to chronic stress and trauma, forcing the individual to live continuously in a state of heightened threat response, with the brain and body remaining focused on survival. A chronically activated stress response without the possibility of rest and recovery has far-reaching physical, mental, and/or behavioral consequences. There is a well-documented increased risk of posttraumatic stress disorder, anxiety, depression, and suicidality (10–13) as well as substance misuse, sleep disorders, and somatic complaints such as chronic pain, gastrointestinal complaints, and gynecological symptoms (2, 14–16). Individuals who are exposed to violence also show greater loneliness, lower self-esteem, and increased self-harming or aggressive behavior compared to those without this experience (17, 18). Risks and negative consequences increase in proportion to the extent and severity of violence, and social trust and well-being decrease (17), possibly impairing their ability to cope with the effects of IPV and reducing the inclination to seek help (18).

As with other traumatic experiences, individuals exposed to IPV follow different trajectories: Some succumb to pathological conditions, others achieve partial or full recovery, and some develop increased functioning over time, with an improved capacity for recovery, resistance, and reconfiguration of their life situations (19). These processes are captured within the concept of resilience, which is grounded in the capacity to regulate emotions, adapt thinking, and adjust behavior in response to changed circumstances. These capacities are shaped by physical and genetic predispositions, personal psychological resources, and social factors that together influence the ability to cope with stressors and recover from adversity (20).

Resilience develops through interaction with the surrounding environment, and supportive professional relationships can further strengthen it. The underlying resources and capabilities can be enhanced through targeted, individually attentive care and support interventions (20). Person-centered care directs attention toward the individual's capacity to experience health and support based on their unique preconditions, rather than focusing solely on presenting problems or symptoms; this means that care and support are adapted to individual needs, values, and wishes. Person-centered communication and support have been consistently linked to more effective and positively perceived support outcomes across a range of helping contexts (21). The relationship between the individual and the professional who provides care or support plays a prominent role in shaping recovery, sense of dignity, and adherence to care. For individuals exposed to IPV, care and support delivery need to account for how traumatic experiences affect coping ability. Trauma-informed approaches have accordingly received increased attention within nursing and healthcare settings: Professionals who adopt this approach understand how traumatic experiences affect health, life situations, and coping strategies, and they design care around the individual's narrative, strengths, and resources (22). These principles extend beyond nursing and healthcare settings: Trauma-informed social work similarly incorporates core principles such as safety, trust, and collaboration into the helping relationship, showing how they apply across different professional roles and care and support settings (23).

Given the high global prevalence and immeasurable long-term and multifaceted consequences of IPV, there is a need for support approaches that actively strengthen individuals' capacity for recovery and resilience in the face of trauma. Further knowledge is needed on how person-centered and trauma-informed approaches to care and support can be applied in practice to promote resilience and support long-term recovery among individuals exposed to IPV. Part of this knowledge concerns not only what care should achieve but also why it works. This review therefore discusses the neurobiological perspective of how trauma shapes threat perception and the capacity to experience safety, and it uses this lens to interpret why person-centered, trauma-informed approaches to care and support matter not only relationally but also physiologically.

The aim of this study was to analyze person-centered care and support interventions aimed at strengthening resilience in adult individuals exposed to IPV.

## Methods

To address the study's aim, an integrative literature review was conducted following Whittemore and Knafl's (24) methodology, which was chosen for its capacity to synthesize both qualitative and quantitative research and thereby capture multiple perspectives on resilience following exposure to IPV. The review followed five stages: problem identification, literature search, data evaluation, data analysis, and presentation of results. In the problem identification stage, the population (adult individuals exposed to IPV), interventions (person-centered care and support), and outcomes (resilience and recovery) were defined to guide the subsequent search and selection. Themes and subthemes were derived inductively from the included studies rather than from predetermined hypotheses (24).

### Search strategy

A systematic literature search was conducted in CINAHL and APA PsycInfo in March 2026. Each database was searched twice, using overlapping but distinct combinations of search blocks, to ensure comprehensive coverage of the relevant terminology (see Supplementary Table 1 for full search strings and hit counts). The search blocks were built around four core concepts: (1) intimate partner violence (e.g., “domestic violence,” “intimate partner violence,” “IPV victim^*^,” and “IPV survivor^*^”); (2) resilience- and recovery-related outcomes (e.g., resilience, empowerment, recover^*^, and rehabilitation); (3) care- and treatment-related approaches, including person-centered and trauma-informed care (e.g., “person-cent^*^ care,” “trauma informed care,” and “trauma sensitiv^*^”); and (4) nursing (nurs^*^, “nurs^*^ care^*^,” “nurs^*^ treatment^*^,” and “nurs^*^ approach^*^”). The nursing block was applied as a required term across all searches, reflecting the review's origin as a nursing master's thesis project. As a result, article retrieval was anchored in nursing-related terminology, which is narrower in scope than the study's aim of examining care and support interventions across professions more broadly. Nonetheless, several included studies involving contributions from social workers, advocates, and other IPV specialists alongside nursing staff, and the review's synthesis therefore reflects person-centered care and support interventions more broadly, consistent with the study's aim. Studies addressing exclusively non-nursing professional roles may not have been captured by this search strategy, which is acknowledged as a methodological consideration.

The inclusion criteria comprised adult individuals (≥18 years) explicitly exposed to IPV, described in qualitative or quantitative, English-language, peer-reviewed studies published between 2015 and March 2026, with a focus on person-centered care and support interventions aimed at strengthening resilience. One included study reported a sample age range of 15–59 years, which exceeds the adult-only inclusion criterion. This study was nonetheless retained because outcome data were not disaggregated by age, so excluding it would have discarded findings relevant to the study's predominantly adult sample rather than isolating the minority of younger participants. This deviation from the protocol is further acknowledged in the Limitations section.

### Study selection

Selection was conducted stepwise, with search results reviewed by title, abstract, and full text (25). Titles were assessed against the study's aim and inclusion criteria, with secondary sources such as meta-analyses, scoping reviews, and narrative reviews excluded, as the focus was on primary studies. Relevant abstracts were then assessed, followed by full-text reviews of the remaining studies to confirm that all inclusion criteria were met. The selection process was documented as a PRISMA flow chart (Figure 1). Title and abstract screening was conducted jointly by CA and EA, followed by independent full-text review and selection by each, with any disagreements resolved through discussion between the two authors and the supervising author (NK).

**Figure 1:**
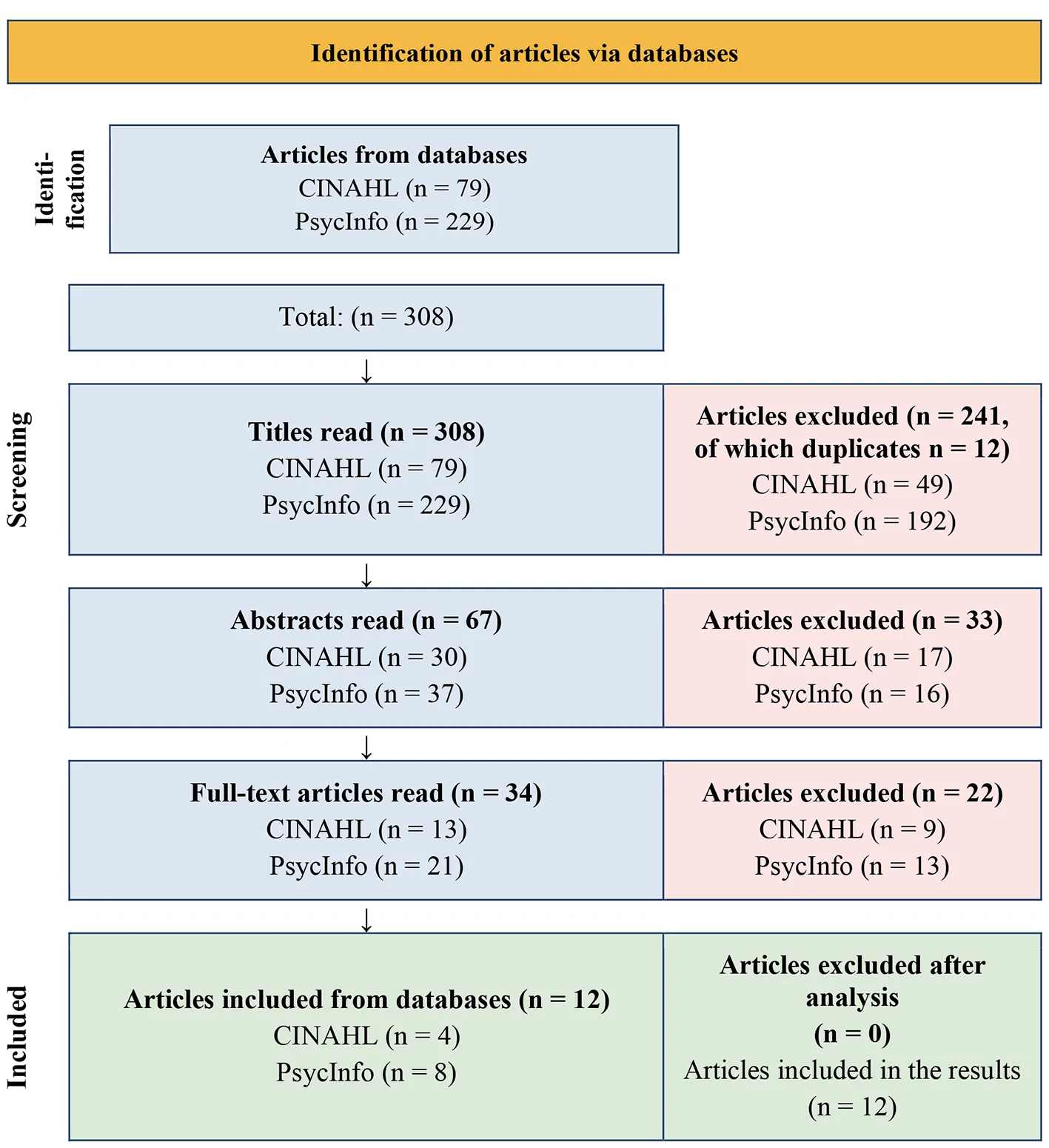
PRISMA **flow chart**. Flow diagram adapted from the PRISMA 2020 statement. A total of 308 records were identified across CINAHL (*n* = 79) and APA PsycInfo (*n* = 229). After removal of duplicates and title screening, 67 abstracts were reviewed, followed by full-text reviews of 34 articles. Twelve articles (4 from CINAHL, 8 from APA PsycInfo) met all the inclusion criteria and were thus included in the analysis.

Study quality was assessed using the CASP Checklist (26): the CASP Qualitative Research checklist was applied to the nine qualitative studies and to the mixed-methods study (a sequential, explanatory design with a nested quantitative subsample), and the CASP Randomized Controlled Trial checklist was applied to the two quantitative studies, one of which employed a quasi-experimental design. Eleven studies were assessed as high quality and one as medium quality, the latter reflecting an increased risk of selection bias due to a quasi-experimental design in which participants self-selected into the intervention or control groups; this study was nonetheless appraised using the CASP RCT checklist, as no CASP tool specific to quasi-experimental designs exists.

### Data analysis

The data were analyzed in five steps: data reduction, data visualization, data comparison, conclusion drawing, and verification (24). In data reduction, all included studies were read repeatedly with a focus on results; findings that were relevant to the study's aim (e.g., which interventions strengthened capacity for recovery and empowerment or how the care relationship affected resilience) were extracted and condensed into meaning-bearing units and then aggregated into codes to enable comparison across studies with differing methodological approaches. In data visualization, the reduced material was organized into an analysis matrix (authors, design, sample, aim, central findings, and preliminary codes) used to identify patterns, similarities, differences, and divergent results. In data comparison, the findings were continuously compared using the principle of constant comparison; recurring codes were grouped into preliminary categories and developed into overarching themes, with attention to variation across contexts, populations, and study designs. In conclusion drawing, the identified themes were synthesized to interpret their combined meaning, focusing on processes, barriers, enabling factors, and connections between themes. Regarding verification, the themes were checked against the included studies to confirm that the interpretations corresponded with the underlying results; contradictory findings and alternative interpretations were considered throughout to strengthen credibility and support a transparent, systematic synthesis (24).

### Reflexivity

The authors' pre-understanding was shaped by their complementary professional backgrounds. CA and EA's pre-understanding was grounded in nursing competence developed through education and clinical experience, including within mental health nursing. NK's pre-understanding was grounded in academic research within psychiatry and neurobiology, combined with ongoing work training support organization staff in trauma-adapted yoga and body-based methods for individuals exposed to IPV. AS's pre-understanding was grounded in academic research within epidemiology and neurobiology. This combination of clinical, psychiatric, neurobiological, epidemiological, and applied support-practice perspectives was considered a resource, contributing complementary subject knowledge while representing a potential source of interpretive bias. To manage this potential bias, the authors engaged in continuous reflection and discussion throughout the research process, using their differing perspectives to challenge and cross-check interpretations.

### Ethical considerations

Of the 12 included studies, 11 had been ethically reviewed and approved by an independent ethics board in the respective country; one was assessed by an institutional ethics board, which determined that formal approval was not required (27). The authors analyzed the studies both individually and jointly to reduce the risk of misinterpretation.

Given that the population under study is particularly vulnerable, studies were required to demonstrate adherence to established ethical guidelines, including the principles of the Declaration of Helsinki: voluntary and informed consent, the right to withdraw, protection against harm, and independent ethical review (28). Particular weight was given to whether the participants had been offered support or referred to assistance in the event of psychological distress. Data protection was a central consideration, as participants shared sensitive personal information; most studies described measures such as anonymization or pseudonymization, secure data storage, and restricted access to material. The studies conducted within Europe adhered to the General Data Protection Regulation (GDPR), while those from other regions applied corresponding local legislation and ethical guidelines.

## Results

### Study characteristics

In total, 12 studies representing eight geographic settings —Australia, Ireland, Iceland, Japan, Puerto Rico, Taiwan, Turkey, and the USA—were included in the quality assessment. Across the studies, 544 individuals with personal experience of IPV were represented (537 women and 7 men). The reported ages across the studies ranged from 15 to 73 years. The reported study means clustered predominantly in the mid-30s to mid-50s, with two studies focused specifically on older women (Halldorsdottir (29): 42–68 years; Irene-López & Kalmakis (30): 62–73 years). One study's reported age range extended below the review's inclusion criterion of 18 years (27); this deviation is addressed in the Limitations section. In addition to the perspectives of individuals exposed to IPV, the results incorporated experiences from 31 professionals working in IPV field (31, 32), hereinafter referred to as care and support staff, spanning nurses, midwives, physicians, psychiatrists, psychologists, social workers, psychotherapists, and IPV advocates. A full overview of the 12 included studies (author, year, country, study aim, design, sample, and main results) is provided in Table 1.

**Table 1:** Overview of analyzed literature.

References	Problem area and aim	Design and method	Sample and study group	Main results	Quality appraisal
**Bahadir-Yilmaz and Öz**. **(****37****)**	Domestic violence (physical, psychological, and economic) has serious negative effects on women's mental health, including reduced self-esteem and feelings of helplessness. **Aim:** to examine the effect of an empowerment program on increasing self-esteem, learned resourcefulness, and coping mechanisms among women exposed to domestic violence	Quantitative, Experimental study with pre-, post-test control group, and follow up design (RCT): A structured group program comprising 10 weekly sessions for intervention group. Measures: • The Personal Information Form (sociodemographic information), • The Coopersmith Self-Esteem Inventory (SEI) • The Rosenbaum's Learned Resourcefulness Scale (RLRS) • The Ways of Coping Inventory (WCI)	Literate women recruited from obstetrics and gynecology departments of the Giresun Maternity Hospital, and at the Family Counseling Center. 30 women in an intervention group and 30 women in a control group. (*N* = 60). Intervention group had a higher mean age (40, 7 ± 11 years vs. 34.6 ± 11.4 years in the control group). No significant difference in the duration of experiencing violence. 100% of the intervention group reported physical and psychological violence that differed, but not significantly from the control group	• Significant improvement: after the program, the intervention group scored significantly higher on self-esteem and learned resourcefulness than the control group. • Improved coping: the intervention group used more optimistic, self-confident and social-support -seeking coping strategies, while helpless and yielding approaches decreased significantly. • Conclusion: the empowerment program was effective in strengthening women's psychological resources and ability to cope with stress after exposure to violence.	**High**
**Danitz et al**. **(****31****)**	Intimate partner violence is a global health problem, and female veterans in the USA are a particularly vulnerable group. There is an urgent need for brief, patient-centered, feasible and acceptable interventions that can be implemented in large health systems **Aim:** to integrate providers' perspectives into the development of the RISE (Recovering from IPV through Strengths and Empowerment) intervention	Qualitative design. Semi-structured, key-informant telephone interviews. Data analyzed using rapid content analysis with hybrid inductive-deductive approach	Veterans' Health Administration providers who worked with female IPV patients. 23 informants (mean age 42.6 years), including psychologists, licensed therapists, social workers, primary care physicians, psychiatrist, experts in intimate partner violence. 2–25 years of work experience with IPV offers. 91% identified as women	• Flexibility: Providers praised RISE's existing modular structure, which allows women to select session topics based on their own needs rather than following a fixed sequence. • Usability: Providers found the manual's structure, conversational scripts, and language user-friendly and easy to use as-is; they additionally requested supplementary materials to further support use in practice. • Clinical integration: Providers identified limited time, resources, and space in busy clinical settings (particularly primary care) as barriers to delivery. • Empowerment focus**:** Providers affirmed that RISE's empowerment-based, non-judgmental stance, emphasizing patient autonomy and choice, was well-matched to the needs of women experiencing IPV, given that autonomy is often what abusive relationships take away.	**High**
**Halldorsdottir**, **(****29****)**	Intimate partner violence (male intimate terrorism) is a serious and escalating problem that can be fatal. There is limited knowledge of how the violence develops and is experienced. **Aim:** to explore the meaning, development, and consequences of partner violence from women's perspectives	Qualitative, phenomenological design. Data collected via 1-3 times in-depth 16 interviews and analyzed using interpretive thematic analysis	9 women (42–68 years, average 57) who had previously been exposed to prolonged violence in an intimate relationship (Divorced > 1 year) (5–32 years of experience, on average 12 years). None of the women had remarried or lived with a partner at the time of the study. All the perpetrating men were the women's former husbands. The women had 2–4 children	• Women experienced escalating violence and severe psychological breakdown. The violence developed through five phases (capture, silencing, death, awakening, and recovery): • Capture: the relationship begins with intense attention experienced as positive but shifts into control and criticism; the woman becomes confused and adapts to avoid conflict; early signs of violence are often denied at this stage. • Silencing: the man uses psychological abuse, threats, and isolation to control the woman; her voice, self-esteem, and agency are lost; feelings of fear, shame, and dependency emerge. • Death phase: violence escalates and becomes life-threatening; the woman feels dehumanized; life is characterized by constant terror and powerlessness. • Awakening: the woman begins to regain feeling and insight into her situation; an inner or outer turning point leads to a decision to leave the relationship; risk increases at separation. • Recovery: a long process marked by trauma and fear but also gradual healing; the woman regains strength and independence; the striving for a new life becomes central.	**High**
**Hargrave et al**. **(****34****)**	Nearly half of all women in the USA experience intimate partner violence during their lifetime. Despite screening recommendations within healthcare, there is limited knowledge of which clinical practices and policies actually help or hinder survivors when seeking support **Aim:** to understand which clinical practices and policies are beneficial, harmful, or insufficient in supporting survivors of intimate partner violence within healthcare, based on their own perspectives	Qualitative interview data from a longitudinal, mix-method study design of the Aspire to Realize Improved Safety and Equity (ARISE) evaluation In-depth, semi-structured interviews (60–90 min). Data analyzed using thematic analysis	A purposive sample of patients from San Francisco's healthcare system. 45 women (18–64 years, mean age 43) who had experienced intimate partner violence within the past year. 36% identified as Black or African American 24% identified as White 13% identified as Multiracial 27% identified as Hispanic or Latinx All were cis-gender women, 98% with male partner	• Relationship-building: trusting, long-term relationships with providers facilitated disclosure of violence, while lack of continuity was a barrier. • Respect for autonomy: providers respecting the woman's choices and pace was crucial; pressure or judgmental attitudes hindered help-seeking. • Systemic barriers: time constraints during visits and fear of mandatory reporting (to police or social services) were major barriers. • Systemic facilitators: visible IPV resource information throughout the clinic and co-located, on-site community IPV advocates who could connect directly with patients were experienced as validating and trust-building.	**High**
**Hou et al**. **(****39****)**	Previous research on interventions for women exposed to violence has primarily focused on reducing psychological problems. There is a lack of knowledge on how group interventions can affect and strengthen women's psychological strengths and inner resources **Aim:** to examine the effects of an 8-week strengths-based support-group intervention on levels of hope, resilience, and depression among Taiwanese women who left a violent intimate relationship	Quantitative, prospective, longitudinal (post-test at 8 weeks and follow up test at 12 weeks) quasi-experimental design. Intervention: 8-week strengths-based perspective group intervention Measures: •The Chinese version of the State Hope Scale (SHS-C) •25-item Resilience Scale (RS-C) •Taiwanese Depression Questionnaire (TDQ)	Women recruited via a domestic-violence prevention center in southern Taiwan. 24 women completed the 12-week study (8 participants in intervention and 16 in control group). No significant differences between the groups in age (30–48 ages, mean age 40), education level, marital status, occupation, economic conditions, number of children, types of abuse, or duration of IPV (average 6 years)	•Limited effects on hope and resilience: overall hope and resilience scores did not differ significantly between groups; only the pathways component of hope showed a significant between-group improvement at week 8. Resilience scores were not significantly affected by the intervention. • Reduced depression: depression levels decreased significantly in the intervention group over time though the between-group difference was not statistically significant. • Conclusion: a strengths-based support group intervention showed some promise for reducing depressive symptoms and improving hopeful thinking (specifically the pathways component) in women who left violent relationships, but effects on resilience were not demonstrated, and findings should be interpreted cautiously given the small, non-randomized sample.	**Medium**
**Irene-López and Kalmakis** **(****30****)**	Intimate partner violence is a global health problem, but there is a lack of knowledge on how older women experience it, particularly within specific cultural contexts such as Puerto Rico. Older women tend to under-report violence, and nurses often lack training to identify violence in this age group **Aim:** to explore the experiences of intimate partner violence among a group of Puerto Rican women over the age of 60, and to understand how the violence has affected their health	Qualitative design, two in-depth interviews with each woman (the second one giving possibility for reflections). Phenomenological analysis by Colaizzi (1978)	Women were recruited from traumatology center of Puerto Rico and a general medicine office. 6 women (62–73 years) (2 married; 2 divorced, 2 separated) who had experienced intimate partner violence after age 60, or who had lived in a violent relationship that continued into older age. None of the participants were employed. All had children (who witnessed IPV)	• Enduring abuse across the lifespan: participants described childhood adversity (including witnessing IPV) alongside physical, psychological, and economic abuse that continued or intensified after age 60. • Cultural silence: cultural norms around machismo and family privacy discouraged disclosure, reinforcing prolonged, hidden suffering. • Lasting health impact: women reported anxiety, depression, insomnia, and one nervous breakdown requiring hospitalization, alongside physical conditions including thyroid disorders, bladder/uterine complaints, and hypertension. • Active seeking of support: women eventually turned to clergy, physicians, nurses, psychiatrists, and police, though support was inconsistent and often only sought during acute crises rather than as ongoing care. • Escape and reflection: most participants ultimately left the relationship and, in retrospect, urged other women not to wait or stay silent.	**High**
**Lewis-O'Conner and Chadwick** **(****27****)**	Despite intimate partner violence being a major public health problem, the patient's own voice is often missing from the design of healthcare policies and practices. There is a risk that care is not sufficiently centered on patients' actual needs, and that important factors for healing are overlooked **Aim**: to engage patients affected by intimate partner violence in order to understand their experiences of care and to use their insights to inform and improve clinical practice and policy	Qualitative survey. Patients were asked to answer three open-ended questions in regard to their healthcare experience. Individuals who were eligible for evidence collection after sexual assault (< 5 days) were asked two additional questions. Qualitative content analysis. Focus group interviews divided across three groups	*N* = 310 (304 women and 6 man). Ages between 15–59 years. IPV (*n* = 100), sexual assault (*n* = 245), and human trafficking (*n* = 14)	• The need for safety: Feeling physically and emotionally safe during care was strongly associated with patient satisfaction. The overwhelming majority of patients described feeling safe and cared for, consistent with a trauma-informed approach. • Non-judgmental approach: Being believed and met with empathy rather than blame was decisive for the patient's experience. • The importance of choice: The patient being given control over their own care process and not being pressured into decisions. • Continuity of care: Patients valued seeing the same care provider over time in order to build trust, while being passed between many different providers and having to repeat their story multiple times, was one of patients' most consistent complaints and a source of distress. • Conclusion: Centering patients' own accounts of care revealed that relational and procedural factors (being believed, retaining choice, and experiencing coordinated rather than fragmented care) shaped their sense of safety and recovery, underscoring the value of person-centered nursing approaches in supporting resilience after intimate partner violence and gender-based violence.	**High**
**Page et al**. **(****38****)**	Intimate partner violence has long-lasting effects on women's mental health, but there is little research on how long-term support interventions affect recovery, particularly for Latina women who may be especially vulnerable due to cultural factors **Aim:** to describe Latina women's experiences of participating in a nurse-led support group (NLLTSGs) for survivors of intimate partner violence	Qualitative design. Individual semi-structured interviews. Data analyzed through a two-phase coding process (line-by-line and focused coding)	Women who participated in support groups in the Houston area. 49 Latina women (26–73 years, mean age 44 years). On average they lived in an abusive relationship 10.47 years. Number of children varied between 0 and 10. Participants had been part of the support group from 2 months up to 9 years. 94% were immigrants from Mexico. 57% were divorces or separated. 43% were unemployed and 6% were students	• Awakening: women moved from confusion and not knowing where to turn, to recognizing that they had been victims of abuse and that change was possible. • Transformation: an inner journey from fear and low self-esteem to hope, confidence, and a new identity as a survivor. • Community: the experience of not being alone; the group offered a safe place to share their stories without being judged. • Empowerment: gaining tools and strength to take control of their lives, set boundaries, become financially independent, and speak up for themselves. • Advocacy: a wish to help other women in similar situations by sharing their story and breaking the silence	**High**
**Reeves and Humphreys** **(****33****)**	Women who have experienced violence have an increased risk of negative health effects. These can be worsened if the care encounter is experienced as traumatic, which can hinder help-seeking. There is a lack of knowledge on how women engage with care and which strategies they use **Aim:** to develop knowledge of sexual or intimate partner violence-exposed women's experiences of healthcare and the strategies they use to navigate the care system	Qualitative design. Semi-structured individual interviews. Analysis conducted using content analysis (Atlas.ti software)	Participants were recruited via advertisements and local support organizations. 14 women (22–63 years, mean age 40.79) who reported and sought support for experience of sexual, physical or emotional intimate partner violence. 71% (9) identified as White and 29% (four) as African American participants. 12 participants reported experiences of physical violence, and all participants reported experiences of emotional abuse	• Diversity of trauma: participants often had complex histories involving different types of violence throughout life. • Immediate care experiences: experiences during acute visits directly after a trauma influenced attitudes toward future care. • Sensory and emotional triggers: situations resembling past abuse could involuntarily recall traumatic memories during care. • Power dynamics: imbalanced provider–patient power undermined trust and was experienced as a separate, compounding difficulty alongside trauma triggers. • The provider's role: the provider's ability to listen, give control, and show empathy was decisive for whether the experience was positive or negative. • Patient strategies: women used active strategies such as carefully choosing providers (often female), preparing mentally, or bringing a support person	**High**
**Roberts et al**. **(****35****)**	People who have experienced sexual assault or intimate partner violence often face significant barriers in encounters with healthcare, which can lead to re-traumatization. There is a need to understand how a trauma-informed approach affects patient engagement and experience of care in a clinical setting **Aim:** to explore patients' experiences of trauma-informed care provided by nurses in primary care to individuals affected by sexual violence or intimate partner violence, and how this care affects engagement, patient experience, and barriers to care	Qualitative design using a naturalistic inquiry approach. Semi-structured individual interviews. Data analyzed using reflexive thematic analysis	Participants were recruited from a local healthcare service actively working with trauma-informed principles. 6 participants, five women and one man (19–72 years, mean age 38), who had experienced sexual violence or intimate partner violence and had completed their full episode of care from nurses	• Strengthened engagement in care: participants felt more motivated to engage in their care when met with a trauma-informed approach prioritizing safety and choice. • Shared burden—the experience of trauma-informed nursing: nurses were described as empathetic and warm; by building strong relationships, patients felt they were not alone with their trauma, creating a sense of emotional safety. • The nurse's role in breaking down system barriers: nurses acted as advocates who helped patients navigate the care system and gave them the confidence to attend appointments and access services they otherwise wouldn't have	**High**
**Saint Arnault and O'Halloran** **(****36****)**	There is substantial research on the immediate structural barriers to leaving a violent relationship, but a lack of knowledge on the sociocultural barriers that prevent women from healing after achieving safety. Rural women in particular are an understudied group **Aim:** to use mixed methods to understand the help seeking and healing trajectory of rural Irish women years after leaving an abusive relationship, and to examine barriers to help-seeking	Sequential, explanatory mixed-method design with a nested quantitative subsample. Qualitative component: semi-structured interviews including life timelines and body maps to visualize symptoms; analyzed using thematic analysis. Quantitative component: surveys measuring symptoms such as depression, anxiety, and physical symptoms (including sleep, gastrointestinal problems, pain, and cardiac and neurological symptoms), Sence of Coherence, barriers to seeking care for trauma, as well as social context, support and social conflict	Women previously exposed to intimate partner violence/domestic violence. 21 women (34–65 years, mean age 45, 5 years) living in rural Ireland who lived in abusive relationship on average over 10 years (range 3–20) and had left their abuser on average 5 years earlier (range 2–10 years)	• Persisting trauma: despite many years having passed, women still experienced high levels of PTSD, depression, and anxiety; they described a sense of being ‘frozen' in their trauma. • Family rejection and enforced silence: rather than finding support after leaving, several women described being rejected or pressured by their own families to return to the abuser; shame and fear of judgment led many to keep their situation private. • Sociocultural barriers over structural ones: quantitative analysis found no relationship between structural barriers (cost, availability, transportation) and symptoms or support, but sociocultural barriers were significantly associated with higher symptom burden and lower social support. • Healing as a journey: healing was not identified as a linear process but as a journey requiring breaking social isolation and as reflected in the study's proposed “unfreezing” model of trauma-informed care	**High**
**Umeda et al**. **(****32****)**	In Japan, healthcare responses to intimate partner violence are limited, partly due to cultural norms prioritizing family cohesion and marginalizing women's experiences of violence. There is a lack of knowledge of how to design care adapted to this context **Aim:** to identify the core values and practices guiding healthcare staff and advocates in their work with women exposed to violence, to inform future training and interventions	Qualitative design. Semi-structured interviews. Data analyzed using thematic analysis according Ryan & Bernard, 2,000	8 professionals who encounter people exposed to intimate partner violence in their work (including midwives, nurses, social workers, and psychotherapists) with average 13.5 years of experience	The study identified two broad themes on how expert professionals approach care for women experiencing IPV: • Guiding principles for care: supporting the woman's autonomy over her own life and decisions, recognizing that recovery follows a unique and non-linear trajectory for each woman, building an equal (non-hierarchical) relationship between provider and patient, and maintaining awareness of the limits of a professional's role in a woman's recovery. • Attitudes and behaviors in practice: trusting and believing what women disclose, respecting their choices and feelings without judgment, waiting patiently for change without pressuring, and engaging with sincerity and genuine concern	**High**

Overview of the studies included in the integrative review, presented by author/year/country, problem and aim, design and method, sample and study group, main results, and quality appraisal (CASP).

In the analysis, three main themes were identified: “A safe, trusting, and accessible care relationship as a foundation for resilience,” “Empowerment through participation, autonomy, and reclaiming control,” and “Mobilization of individual and social resources for long-term recovery.” Based on these three main themes, seven subthemes were formed.

Together, these themes illuminate the complexity of the approach to and provision of care and support for adult individuals exposed to IPV, with a particular focus on person-centered interventions that strengthen the individual's resilience. The results are presented based on the identified themes and associated subthemes (Table 2).

**Table 2:** Three identified main themes with seven associated subthemes.

Main theme	Subtheme
A safe, trusting, and accessible care relationship as a foundation for resilience	Approach, trust, and being listened to
	Being given a voice in one's own care
	Structural and sociocultural barriers and enablers
Empowerment through participation, autonomy, and reclaiming of control	Shared decision-making and reclaiming of control
	Empowerment as a process for resilience
Mobilization of individual and social resources for long-term recovery	Strength-based interventions and coping development
	Social support, community, and collective resilience

### A safe, trusting, and accessible care relationship as a foundation for resilience

This main theme captures how interpersonal, organizational, and sociocultural factors interact within the care encounter. A functioning care relationship depends on more than approach alone; accessibility, continuity, coordination, and the extent to which care addresses stigma and norms all play a part. Together, these elements create the conditions for safety, participation, and long-term recovery. Where any one element is missing, help-seeking can be impeded and resilience more difficult to build.

#### Approach, trust, and being listened to

The analysis showed that how care was approached made the difference for individuals exposed to violence, shaping their sense of safety, their trust, and whether they were willing to seek and continue care (33, 34). Years after the violence has occurred, care encounters can bring traumatic memories back to life. Sensory impressions, such as touch and medical examinations, and emotional cues, such as body language or certain conversations, can trigger strong discomfort and a sense of reexperiencing the trauma (33).

Poor communication, the absence of informed consent, and experiences of not being believed or being diminished deepened vulnerability and eroded trust in care (27, 33). An approach built on empathy, respect, and active listening did the opposite: It let the individual feel seen and affirmed. Being able to tell their story at their own pace, without interruption, eased feelings of loneliness and gave the individual strength, which in turn drove their willingness to seek and continue care (30, 34).

Performing empathetic actions, noticing concern, providing clear information about what to expect during the examination, and letting the individual retain control over what was and was not permitted during physical examinations eased stress responses and built a stronger sense of safety (33). Above all, being listened to made the care relationship work. When staff let the individual set the pace without pushing for decisions, trust grew and, with it, the willingness to open up about the violence they had experienced (32).

Trust was also built through respect, reliable communication, and staff's genuine understanding of what the individual had been through (35). A trauma-aware approach, whereby staff recognized the impact of trauma and emphasized that the violence was not the individual's fault, eased shame and increased both safety and engagement in care (34, 35), serving as a counterweight to the familial and social rejection that otherwise pushed individuals into silence and self-blame (36).

#### Being given a voice in one's own care

The studies showed that having a voice in one's own care, within a relationship the individual could trust, was a precondition— not an optional extra—for a supportive care encounter (34). Individuals wanted a say in decisions about their own care. A standardized, paternalistic approach felt violating and, in some cases, drove people away from care altogether (34). Particularly damaging were situations in which individuals felt pushed toward treatment or decisions without their specific situation being taken into account (33, 34). Flexible, individually adapted care, whereby needs and preferences were actually considered, did the opposite, creating a stronger sense of control, empowerment, and safety (35). Some individuals took autonomy into their own hands, developing strategies such as seeking out a provider whom, based on their experience, they deemed safe and respectful. A preference for female providers was evident, especially for physical examinations, and was tied to previous experiences of violence (33). Being provided with clear information about their care options and available resources, being listened to, and having a real say in decisions strengthened both autonomy and trust in the care relationship.

#### Structural and sociocultural barriers and enablers

Structural and organizational factors often heavily influenced the kind of care and support that individuals exposed to violence were able to access. Limited time with staff, long waiting lists, and high staff turnover made trusting care relationships difficult to build. Traumatic experiences then had to be retold repeatedly, resulting in a burden that could be re-traumatizing in itself (27, 34).

Care could also feel fragmented and difficult to navigate. Support that came only in crisis moments, rather than as ongoing, coordinated care, left individuals to navigate help largely on their own (30). However, where healthcare and community-based organizations worked together, integrated and coordinated care built more trust and a more positive overall care experience (34).

Beyond the care system itself, sociocultural norms, shame, and stigma stood in the way of help-seeking. Norms around gender roles, family, and privacy normalized violence and kept it invisible, leaving many to stay silent, remain in the relationship, or feel that the situation was somehow their own fault (30, 36). Against this backdrop, a culturally aware, nonjudgmental approach within care made it possible to lower these barriers and open the door to support and recovery, as reflected in Saint Arnault and O'Halloran's (36) trauma-informed nursing practice model, which frames 'unfreezing', alongside resolving shame and confronting stigma, as central to supporting healing from the isolation and silence that can persist for years after the individual leaves an abusive relationship.

As our analyses show, being heard, trusted, and met without judgment were central to how a care relationship could become a foundation for resilience in individuals exposed to IPV. Where flexibility, respect for the individual's own priorities, and providers who could be trusted to act in their interest were present, agency grew, along with confidence in one's own capability and a stronger sense of the future. Where these elements were missing, powerlessness could deepen instead, becoming another obstacle to resilience and long-term recovery.

### Empowerment through participation, autonomy, and reclaiming control

This main theme brings together findings that describe recovery after IPV as a successive reclaiming of control, whereby participation in one's own care and recovery, autonomy, and empowerment function as both the process and goal in building resilience.

#### Shared decision-making and reclaiming control

The studies showed that autonomy and active participation in one's own care were preconditions—not optional extras—for a supportive care encounter (34). Both care and support staff and participants described shared decision-making, flexible goal setting, and respect for the individual's own recovery pace as central to support interventions (31, 32, 34). What mattered was giving the individual space to define what was most important in their own situation, whether that concerned safety, children, finances, relationships, mental health or any other consideration. Providers saw a flexible, individually adapted design, letting women (the population both studies focused on) choose session content based on their own priorities, as a good fit for women's autonomy and choice (31), echoing practitioner views on the need for individualized recovery timelines (32). When individuals felt pressured or steered toward specific decisions, their trust in their own judgment decreased, feeding into their feelings of powerlessness and loss of autonomy. Freedom of choice, transparency, and collaboration stood out as the principles that kept care from repeating past experiences of losing control (27, 32, 34). The providers themselves reinforced this: An empowerment-based, nonjudgmental, autonomy-centered approach fit the needs of women exposed to IPV particularly well precisely because autonomy is so often what is taken away in abusive relationships (31).

#### Empowerment as a process for resilience

Empowerment-based interventions improved individuals' self-esteem, coping, and resourcefulness in the face of stress (37). However, empowerment was not merely an intervention goal; the findings pointed to it working as a process in its own right, unfolding over the course of recovery.

Structured empowerment programs and reflective conversations with care and support staff gave individuals room to identify problems, weigh alternative courses of action, and build their own strategies for managing their situation, as reflected quantitatively in the statistically significant improvements in their self-esteem and learned resourcefulness following a structured empowerment program (37). Qualitative accounts pointed to a similar shift playing out over time, with individuals describing a changed self-image, moving from feeling vulnerable to feeling capable of acting on their own behalf, and becoming increasingly confident that they could shape their life situation (38). This process of making visible one's own strengths, past survival strategies, and personal resources came through across both quantitative and qualitative findings as central to building a sense of capability, hope, and agency (37, 38).

The results of our analyses strengthen the case that person-centered care and support interventions build resilience in adult individuals exposed to IPV when they enable participation in their own care and recovery, support autonomy, and help individuals reclaim control. Interventions designed collaboratively with the individual gave them back a sense of agency, built their confidence in their capabilities, and supported long-lasting recovery.

### Mobilization of individual and social resources for long-term recovery

This main theme captures how person-centered interventions help individuals identify, mobilize, and build on both personal and social resources. Our analyses showed that interventions built around an individual's existing strengths and competencies rather than their weaknesses or problems created the right conditions for a sustainable recovery process, reinforced by coping strategies, trauma-informed guidance, and supportive communities.

#### Strength-based interventions and coping development

Person-centered, strength-based interventions helped mobilize individual resources, coping strategies, hope, and a sense of the future. Interventions that actively identified and built on an individual's existing strengths showed some promise for reducing depressive symptoms and improving hopeful thinking; however, effects on resilience itself were not demonstrated, and these findings should be read cautiously given the study's small, non-randomized sample (39). Comparable interventions elsewhere produced statistically significant improvements in self-esteem and coping ability specifically (37).

Support groups grounded in a strengths-based approach helped individuals progress from seeing themselves as vulnerable to seeing themselves as capable of acting on their own behalf, such as making decisions, setting goals, and seeking support (38). Shifting the focus from symptoms and deficits to strengths and lived experience-built competence, hope, and coping ability. This enabled the individuals to develop coping strategies that actively supported recovery; these included carefully choosing care providers, preparing mentally for care encounters, and bringing a support person along (33) alongside more optimistic, self-confident, and social-support-seeking approaches, with helpless and yielding coping strategies decreasing significantly (37). These strategies worked as protective factors for long-term resilience. In the studies that specifically addressed trauma-informed care, understanding a person's reactions in light of their past trauma emerged as equally important (33, 35).

#### Social support, community, and collective resilience

Social support, community, and relational recovery were central to for long-term recovery. Group-based interventions offered more than emotional support; they also fostered a sense of belonging, recognition, and shared strength.

Long-term support groups and community helped counter isolation, normalize experiences, and fostered hope for a life beyond the violence. Hearing from others in similar situations opened new ways of understanding one's own experience, identifying resources, and setting goals for the future (38). For some, this impact extended even further: Individuals became a source of support for others, in turn strengthening their own self-esteem, sense of meaning, and agency (38). The reverse was also observed: Sociocultural barriers to help-seeking, including shame, hopelessness, feeling ‘frozen,' and mistrust in available support, were statistically significantly associated with greater symptom burden and lower social support, independent of structural barriers such as cost or availability (36), underlining that countering isolation is a necessary step in healing rather than a side effect of it. In other words, social community comprised not only support; it was also a process through which individual resilience could become collective.

Our analyses show that mobilizing individual and social resources was a core part of recovery and resilience building after IPV. Where strength-based approaches, coping-strategy development, and supportive social contexts were all present, agency, stability, and a strong sense of the future followed. Where these were missing, access to resources narrowed instead, rendering resilience and long-term recovery more difficult to achieve.

## Discussion

This review synthesized just over a decade of research on person-centered care and support interventions that strengthen resilience in adult individuals exposed to IPV, summarized in three overarching themes: the importance of a safe, trusting, and accessible care relationship as the foundation for strengthening resilience; empowerment through active participation in one's own care, through the establishment or reestablishment of autonomy, and the knowledge that control can be reclaimed; and the necessity of mobilizing both individual and social resources for long-term recovery. As the focus of this review was on the process of resilience, we discuss the results below within the contextual frameworks of person-centered and trauma-informed care and support.

### Resilience, the strengthened capacity of recovery, resistance, and reconfiguration as a key concept in effective and long-term care and support

Resilience is not a settled concept in the literature. A recent systematic review of resilience among women survivors of IPV found that process-, trait-, and outcomes-oriented definitions all coexist alongside correlates operating at the individual, relational, and contextual levels (40). Our three themes trace roughly the same architecture—a relationship-level foundation, an individual process of reclaiming agency, and a mobilization of personal and social resources—suggesting that resilience-focused care needs to work across all three levels simultaneously rather than targeting any single one in isolation.

Sense of control stands out particularly clearly across this study's findings as well as other literature: Restoring control has been identified as one of the principal sources of resilience following trauma, as trauma so fundamentally destroys a person's control over their own life (40). Women describing their own resilience have characterized it as a series of shifts (cognitive, emotional, and behavioral) moving toward resistance, a renewed experience of control, and positivity (41). These shifts echo the recovery, resistance, and reconfiguration that define resilience as a construct (19). Empirical work using the resilience portfolio model has found that women's personal strengths are positively associated with both wellbeing and posttraumatic growth—positive psychological change that can emerge from struggling with adversity—after IPV; together, these account for a substantial share of the variance in each (42), underscoring that growth after trauma rests on identifiable, nurture-able resources and not simply on disposition. These findings align closely with ours: When care actively enables the individual to formulate their own goals, choose the areas of focus within interventions and treatment, and set their own pace of recovery, it supports exactly this kind of active reconfiguration of one's life situation. This is particularly central in the IPV context, wherein autonomy, confidence in one's own judgment, and the experience of agency have often been undermined over time. Reclaiming these capacities corresponds with empowerment as a dynamic, context-dependent process that is central to building resilience (43).

At the same time, resilience is not a linear or uniform process. In acute crisis or during pronounced trauma-related stress reactions, a strong focus on self-determination can initially feel overwhelming rather than strengthening; this is a reminder that the capacity for recovery, resistance, and reconfiguration develops at a pace that is shaped by the individual's current resources and situation, not by a fixed timeline (19). The same context dependence also shows up at a broader level: For women experiencing IPV, the same environment can both cultivate and undermine resilience, depending on the resources that it makes or does not make available (44). In this light, resilience is not simply increased self-esteem or individual strength; it is a process whereby the ability to define one's needs, make decisions, and orient toward future goals is shaped by the interplay between individual resources, life situation, and timing; this is consistent with trauma-informed care's emphasis on the gradual rebuilding of control, safety, and individual adaptation (45, 46).

Hope, or its absence, is one of the clearest examples of how individual resources shape the resilience process. Psychological distress, and hopelessness in particular, has been shown to be strongly associated with IPV survivors' engagement in help-seeking, suggesting that hope and future orientation function not only as an outcome of recovery but also as a factor that actively shapes whether a person continues to engage with support at all (47). Addressing hopelessness, therefore, is not a peripheral concern for care and support services; rather, it may be one of the conditions that determine whether resilience-building interventions can effectively reach an individual and be applied by them in practice.

In this context, coping strategies are more than individual skills; they are part of how resilience is actively built and sustained over time, reflecting the ongoing resistance that Lepore and Revenson (19) describe as central to the concept of resilience. However, these strategies do not develop in isolation; they are shaped by access to external resources. Among women exposed to IPV, greater social support has been shown to significantly predict higher resilience, alongside spirituality and fewer violent relationships (48). Factors such as housing and both informal and formal social support have been identified as resources that build resilience alongside individual strategies such as maintaining a sense of control, humor, and personal goals (49). This is broadly consistent with perspectives that emphasize the importance of social networks and available resources in the context of exposure to violence (50, 51). This is consistent with the present findings, which showed that sociocultural barriers and isolation, rather than structural barriers such as cost or transportation, were more strongly associated with symptom burden and reduced social support, underscoring the importance of the social dimension of resilience and recovery. Supportive relationships, networks, and communities enable the exchange of experiences, recognition, and normalization, which can reduce isolation and strengthen the sense of belonging—conditions that allow individual resilience to become more collective and sustained.

### Person-centered and trauma-informed care as crucial components of care and support

Even though person-centered care has often been articulated through a nursing lens, it is not a nursing-specific idea (52). Social work scholars have made a similar case: Authentic person-centered practice depends on the relationship between the professional and the person receiving care, not on the competencies of any single discipline (53). Given that this review's sample of care and support staff spanned nursing, social work, and other IPV-advocate roles, this cross-disciplinary grounding matters; the knowledge base to which our findings point needs to be applied across professions—not remain confined to one.

Trauma-informed care places a specific demand on the professional: They must have an understanding of how trauma actually affects the body physiologically rather than only how it feels to the person describing it. Prolonged exposure to violence keeps the nervous system locked into a sympathetic, threat-oriented state, and this has a direct clinical consequence: A care encounter can reactivate this state just as easily as it can help regulate it (54). A touch during a physical examination, an unexpected question, or even body language that is read as dismissive can be processed by a dysregulated nervous system as a continuation of the original threat rather than as a moment of care. This is not a matter of the person being “too sensitive”; it is how a nervous system shaped by chronic threat has evolved to respond—that is, not merely to react to danger but also to predict it based on all that it has learned. The brain's most basic task is not to react to incoming stimuli but to anticipate them; it builds a continuous internal model of reality from prior experience and incoming sensory information, and uses this model to anticipate what is about to happen before it fully arrives (55). Central to this process is precision—the brain not only registers a prediction error; it also weighs how reliable this signal is and lets the most trustworthy signals carry the most weight in updating what it expects next.

Trauma changes this weighting. Threat-related signals are assigned disproportionately high precision, even when they are objectively neutral, while safety signals are given too little weight to register as credible (56). A sound, a glance, a bodily sensation that poses no real danger can still be read as a threat because the brain's internal model already assumes that danger is likely. In other words, safety does not automatically feel safe; it has to compete against a system that is already primed to expect harm.

Read through this neurobiological lens, the encounters that our findings describe as re-traumatizing, then, were not failures of empathy in care and support personnel alone; they can be understood as failures to account for what trauma had already done to the person's physiology before they ever walked into the room and for the fact that the brain's primary task is prediction rather than passive reception. On this interpretation, a rushed explanation, an unexpected touch, and a dismissive tone not only feel unpleasant to someone with this history; they also confirm what the nervous system already expected. A single reassuring sentence from staff would not be expected to simply outweigh this, as safety would need to be demonstrated consistently enough and clearly enough to actually shift the precision that the brain assigns to it. This offers one plausible explanation for why our findings show that approach, consistency, and being heard mattered so much not merely as a matter of pleasant bedside manner but, within this framework, as a possible neuronal mechanism by which trust becomes possible again.

Despite this strong neurobiological grounding, trauma-informed care remains inconsistently implemented and under-researched relative to how common trauma exposure is among the people whom the health and social care systems serve (22). Healthcare providers are often not equipped to recognize the signs of trauma or to adapt their practice accordingly, even though they are frequently the first point of contact for people affected by interpersonal violence (57). The same gap appears in social work: Trauma-informed principles are taught inconsistently, and practitioners often lack structured training in how to apply them (58, 59). Beyond clinical settings, trauma-informed principles have also been articulated as an ethical and methodological practice grounded in safety, participation, reflexivity, and attention to power, particularly in research with survivors of violence and displacement (60, 61), underscoring that these principles extend well beyond any single professional or disciplinary context. Building this knowledge into professional education across disciplines rather than within only one is not a peripheral addition to existing training; it is a precondition for the kind of care that individuals actually need according to our findings.

However, knowledge alone is insufficient if the organizational systems around staff work against it. Our findings showed this clearly: Structural factors such as time pressure, lack of continuity, and fragmented care hindered the establishment of trusting care relationships regardless of how trauma-informed any individual member of staff might be, and this pattern is consistent with other research (62). Equal, effective care depends not only on what a practitioner knows but also on whether the organization around them allows their knowledge to be applied through continuity, time, and a working culture that does not shame or blame people (63).

Person-centered and trauma-informed care are not simply techniques that an individual practitioner can apply on their own. By definition, both presuppose an organizational context that actively supports equality, respect, and freedom from paternalism; without this context, the philosophy behind them cannot be realistically put into practice. This is not to say that a paternalistic approach in care is universal or inevitable. Healthcare more broadly has been moving away from paternalistic models, replacing them with approaches built on partnership, shared decision-making, and active patient involvement in the design of care itself through patient advisory councils, structured staff training, and organizational cultures explicitly built to counter paternalistic and hierarchical norms (64). This shift depends on more than organizational structure; it also depends on how individual practitioners understand their own role. Reciprocal, nonhierarchical relationships between provider and survivor, rather than a unidirectional “helping” dynamic, have been identified as central to trauma- and-violence-informed care across a wide range of service settings (65). This kind of humility regarding the limits of what a single professional can do (a theme also found among Indigenous IPV advocates reflecting on their own practice) is not a lesser form of care; it protects against exactly the dynamic that this section has described, wherein good intentions end up reproducing the loss of control that a person has already experienced (66). However, where paternalism does appear, it does more than feel unpleasant; because most people seeking support after IPV are women, it risks reproducing the very gendered power imbalance that the violence itself was built on, deepening powerlessness rather than alleviating it (45). At the same time, the shift away from paternalism is desired in equal measure; preferences for how much decision-making responsibility to take on vary substantially between individuals, and shared decision-making, however desirable in principle, is not universally preferred by everyone to whom it is offered (67). This is a reminder that person-centered care means responding to what a specific person actually wants, not assuming what they should want. Similar dynamics have also been documented in other cultural contexts: In China, for example, formal help-seeking channels have been described as patriarchal and paternalistic in ways in which informal, relationship-based channels are not (68).

Men face a related but distinct barrier: Stigma, norms around masculinity, and difficulty identifying as victims can keep their experiences underrepresented and unaddressed (69, 70). Across both the gendered power imbalance affecting women and the underrepresentation affecting men, stigma, shame, and guilt function as a throughline, standing out as central to why recovery does not happen (71). Taken together, these patterns reflect dynamics that extend beyond individual attitudes or care encounters: In the IPV context, gender operates as a societal structure and not merely as a personal characteristic, shaping what both women and men are expected to tolerate, disclose, or resist (6). Although those who provide person-centered, trauma-informed care cannot undo this structure, they can refuse to reproduce it within the one space they do control: the care encounter itself. None of this means that trauma-informed care is a finished or fully proven framework. Its evidence base remains inconclusive in places, and there is a risk that an intense focus on trauma can end up defining people primarily through it rather than through their capacity to recover (72). What this suggests is not that trauma-informed knowledge is unnecessary but that it needs to work alongside person-centered principles, such as attentiveness to the individual's priorities and pace. Care and support staff have pointed to what this can look like in practice: an empowerment-based, nonjudgmental, autonomy-centered design; this aligns with what women say they need, and it fits this population particularly well precisely because autonomy is often taken away in abusive relationships (31). Understanding the neurobiological and physiological consequences of trauma is necessary but is not sufficient by itself. Knowledge of what trauma does to the brain and body has to be applied in the context of the individual and grounded in genuine attentiveness to the person carrying this history, which is not simply remembered but also actively lived. As this paper has argued, each person's reality is built from the brain's own predictions and shaped by prior experience. No person in this position experiences any single, objective encounter the same way; there is only the reality that each person's brain has learned to expect. At its core, person-centered, trauma-informed care and support is the practice of meeting this reality rather than assuming one's own.

### Strengths and limitations

The integrative review approach (24) that was adopted for this study was a key strength, as it allowed for qualitative and quantitative evidence to be synthesized within a single analysis, which was appropriate given the complexity of resilience as a construct. The search-and-selection process was systematic and transparent, conducted independently and then jointly by the review team, and quality-appraised using CASP across all study designs, thereby strengthening the credibility of the synthesis. The included studies spanned eight countries across four continents and a wide age range. The studies, two of which focused specifically on older women, offered insight into resilience across different life stages. Bringing a neurobiological, predictive-processing perspective into the interpretation of the findings added an additional layer of explanatory depth not typically found in integrative reviews on this topic, grounding the practical relevance of trauma-informed care in an established physiological mechanism rather than principle alone. The review team's complementary backgrounds—spanning nursing, psychiatric research, epidemiology, and applied trauma-informed practice—supported a more comprehensive reading of the material, with reflexive discussion used throughout to limit the influence of individual pre-understanding. Finally, only studies with a documented ethical review or an explicit ethical rationale for which formal approval was not required were included, reflecting the vulnerability of the studied population.

Several limitations should be considered. The search strategy was also restricted to two databases, CINAHL and APA PsycInfo, chosen for their strong coverage of nursing and psychological literature; the exclusion of broader multidisciplinary databases such as PubMed/MEDLINE or Web of Science may have led to the omission of relevant literature from other health disciplines. Most included studies were qualitative, which supports a rich understanding of experience but limits the ability to draw generalizable quantitative conclusions. The sample was heavily skewed toward women (537 of 544 participants), which is consistent with the broader IPV research landscape but limits how confidently the findings can be extended to men's experiences. Most studies were also conducted in high- and middle-income countries, meaning that experiences from lower-income settings are underrepresented. Combining studies with different designs, contexts, and definitions of resilience, which is an inherent feature of integrative review methodology, also makes direct comparison across studies difficult, and, as with any thematic synthesis of qualitative material, some degree of interpretive subjectivity cannot be fully excluded despite the use of reflexive, team-based analysis.

Two further methodological considerations are specific to this review. First, one included study (27) reported an age range extending below the inclusion criterion of ≥18 years; as findings were not disaggregated by age, the exact number of minors in the sample could not be determined. Given that mean ages across the remaining studies indicated a predominantly adult population, this deviation is considered unlikely to have materially affected the findings but is nonetheless noted as a limitation. Second, the search strategy required a nursing-related term in every search combination, reflecting the review's origins as a nursing master's thesis project. Although this ensured a rigorous, reproducible search, it may have excluded studies on person-centered care and support delivered exclusively by non-nursing professionals. That several included studies nonetheless involved social workers and IPV advocates suggests that this risk was limited in practice, but it remains a relevant consideration when interpreting the review's scope.

## Conclusion

This review shows that person-centered, trauma-informed care and support strengthens resilience in individuals exposed to IPV by fostering a trusting care relationship, supporting autonomy and participation in one's own recovery, and mobilizing individual and social resources. Whether this can be realized in practice depends not only on staff knowledge but also on the organizational and structural conditions surrounding care. Care must also remain attentive to gender in a broader sense: Although paternalistic dynamics disproportionately affect women's autonomy over their care, men's experiences of IPV remain comparatively underrepresented and often unrecognized, pointing to a different but equally important gap in how care is influenced by gender. Resilience itself is not linear; it develops through the ongoing interplay between a person's own resources, their relationship with care and support staff, and the wider conditions that shape what care and support services can offer toward recovery and resilience-building. Facilitating this process begins with recognizing that no one's reality is absolute; we can only ever share it with one another through language, which is an inherently limited means of conveying one person's reality to another, a limitation that trauma can compound and that practitioners must with patience and empathy.

## Data Availability

The original contributions presented in the study are included in the article/Supplementary material, further inquiries can be directed to the corresponding author.
