# Supplementary material for: Person-centered, trauma-informed care and support for resilience after intimate partner violence: an integrative review through a neurobiological lens
Source: Front Public Health. 2026 Oct 27;14:1962315. doi: 10.3389/fpubh.2026.1962315 (PMC13748345; doi:10.3389/fpubh.2026.1962315)
Supplement: Supplementary file 1 [file Table_1.docx]

# Supplementary Table 1 — Search Strategies

*Systematic literature searches were conducted in CINAHL and APA PsycInfo. Each database was searched twice, using overlapping but distinct search-block combinations, to ensure comprehensive coverage of relevant terminology. Shaded rows indicate the final limits applied (peer-reviewed, publication years 2015–2026, English language) and the resulting number of hits, titles, abstracts, full texts read, and articles selected for the results.*

## CINAHL 1 — Search date: 2026-03-18

| **Search terms** | **Number of hits** | **Titles read** | **Abstracts read** | **Full texts read** | **Articles selected for results** |
| --- | --- | --- | --- | --- | --- |
| S1 care* | 1,955,319 |  |  |  |  |
| S2 treatment* | 1,455,246 |  |  |  |  |
| S3 "patient* cent* care" | 46,899 |  |  |  |  |
| S4 "person* cent* care" | 4,282 |  |  |  |  |
| S5 "person* cent* approach" | 10 |  |  |  |  |
| S6 "patient* cent* approach" | 1,679 |  |  |  |  |
| S7 "trauma informed care" | 1,424 |  |  |  |  |
| S8 "trauma sensitiv*" | 149 |  |  |  |  |
| S9 S1 OR S2 OR S3 OR S4 OR S5 OR S6 OR S7 OR S8 | 3,039,206 |  |  |  |  |
| S10 resilience | 26,374 |  |  |  |  |
| S11 persistence | 15,663 |  |  |  |  |
| S12 "mental ability" | 155 |  |  |  |  |
| S13 "recovery ability" | 26 |  |  |  |  |
| S14 "mental recover*" | 69 |  |  |  |  |
| S15 resistance | 151,888 |  |  |  |  |
| S16 recover* | 161,225 |  |  |  |  |
| S17 empowerment | 29,887 |  |  |  |  |
| S18 rehabilitation | 224,863 |  |  |  |  |
| S19 S10 OR S11 OR S12 OR S13 OR S14 OR S15 OR S16 OR S17 OR S18 | 569,720 |  |  |  |  |
| S20 "domestic abuse" | 758 |  |  |  |  |
| S21 "domestic violence" | 14,101 |  |  |  |  |
| S22 "intimate partner violence" | 16,967 |  |  |  |  |
| S23 "partner violence" | 17,192 |  |  |  |  |
| S24 "intimate partner abuse" | 248 |  |  |  |  |
| S25 "partner abuse" | 556 |  |  |  |  |
| S26 "relationship violence" | 217 |  |  |  |  |
| S27 "IPV victim*" | 789 |  |  |  |  |
| S28 "IPV survivor*" | 330 |  |  |  |  |
| S29 S20 OR S21 OR S22 OR S23 OR S24 OR S25 OR S26 OR S27 OR S28 | 28,071 |  |  |  |  |
| S30 nurs* | 1,069,139 |  |  |  |  |
| S31 "nurs* care*" | 81,824 |  |  |  |  |
| S32 "nurs* treatment*" | 204 |  |  |  |  |
| S33 "nurs* approach*" | 800 |  |  |  |  |
| S34 S30 OR S31 OR S32 OR S33 | 1,069,139 |  |  |  |  |
| S35 S9 AND S19 AND S29 AND S34 | 85 |  |  |  |  |
| **Limits: peer reviewed, years: 2015-01-01 - 2026-03-18, English** | 44 | 44 | 19 | 7 | 2 |

## CINAHL 2 — Search date: 2026-03-18

| **Search terms** | **Number of hits** | **Titles read** | **Abstracts read** | **Full texts read** | **Articles selected for results** |
| --- | --- | --- | --- | --- | --- |
| S1 nurs* | 1,069,139 |  |  |  |  |
| S2 "trauma informed" | 3,802 |  |  |  |  |
| S3 "gender based violence" | 2,484 |  |  |  |  |
| S4 "domestic violence" | 14,101 |  |  |  |  |
| S5 "intimate partner violence" | 16,967 |  |  |  |  |
| S6 "IPV victim*" | 789 |  |  |  |  |
| S7 "IPV survivor*" | 330 |  |  |  |  |
| S8 "relationship violence" | 217 |  |  |  |  |
| S9 S3 OR S4 OR S5 OR S6 OR S7 OR S8 | 29,317 |  |  |  |  |
| S10 S1 AND S2 AND S9 | 39 |  |  |  |  |
| **Limits: peer reviewed, years: 2015-01-01 - 2026-03-18, English** | 35 | 35 | 11 | 6 | 2 (2)* |

**Two additional article was identified and is counted together with the two articles already selected from CINAHL 1 (total of 4 unique articles from CINAHL).*

## APA PsycInfo 1 — Search date: 2026-03-18

| **Search terms** | **Number of hits** | **Titles read** | **Abstracts read** | **Full texts read** | **Articles selected for results** |
| --- | --- | --- | --- | --- | --- |
| S1 nurs* | 239,605 |  |  |  |  |
| S2 "nurs* care*" | 8,788 |  |  |  |  |
| S3 "nurs* treatment*" | 56 |  |  |  |  |
| S4 "nurs* approach*" | 227 |  |  |  |  |
| S5 S1 OR S2 OR S3 OR S4 | 239,605 |  |  |  |  |
| S6 resilience | 59,271 |  |  |  |  |
| S7 persistence | 29,653 |  |  |  |  |
| S8 "mental ability" | 3,078 |  |  |  |  |
| S9 "recover* ability" | 37 |  |  |  |  |
| S10 "mental recover*" | 64 |  |  |  |  |
| S11 resistance | 58,494 |  |  |  |  |
| S12 recover* | 117,534 |  |  |  |  |
| S13 empowerment | 26,993 |  |  |  |  |
| S14 rehabilitation | 232,386 |  |  |  |  |
| S15 S6 OR S7 OR S8 OR S9 OR S10 OR S11 OR S12 OR S13 OR S14 | 488,837 |  |  |  |  |
| S16 "domestic abuse" | 1,310 |  |  |  |  |
| S17 "domestic violence" | 22,377 |  |  |  |  |
| S18 "intimate partner violence" | 21,024 |  |  |  |  |
| S19 "partner violence" | 21,516 |  |  |  |  |
| S20 "intimate partner abuse" | 519 |  |  |  |  |
| S21 "partner abuse" | 1,795 |  |  |  |  |
| S22 "relationship violence" | 568 |  |  |  |  |
| S23 "IPV victim*" | 457 |  |  |  |  |
| S24 "IPV survivor*" | 552 |  |  |  |  |
| S25 S16 OR S17 OR S18 OR S19 OR S20 OR S21 OR S22 OR S23 OR S24 | 37,516 |  |  |  |  |
| S26 S5 AND S15 AND S25 | 309 |  |  |  |  |
| **Limits: peer reviewed, years: 2015-01-01 - 2026-03-18, English** | 176 | 176 | 19 | 13 | 7 |

## APA PsycInfo 2 — Search date: 2026-03-18

| **Search terms** | **Number of hits** | **Titles read** | **Abstracts read** | **Full texts read** | **Articles selected for results** |
| --- | --- | --- | --- | --- | --- |
| S1 nurs* | 239,605 |  |  |  |  |
| S2 "trauma informed" | 6,697 |  |  |  |  |
| S3 "gender based violence" | 2,215 |  |  |  |  |
| S4 "domestic violence" | 22,377 |  |  |  |  |
| S5 "intimate partner violence" | 21,024 |  |  |  |  |
| S6 "IPV victim*" | 457 |  |  |  |  |
| S7 "IPV survivor*" | 552 |  |  |  |  |
| S8 "relationship violence" | 568 |  |  |  |  |
| S9 S3 OR S4 OR S5 OR S6 OR S7 OR S8 | 38,254 |  |  |  |  |
| S10 S1 AND S2 AND S9 | 65 |  |  |  |  |
| **Limits: peer reviewed, years: 2015-01-01 - 2026-03-18, English** | 53 | 53 | 18 | 8 | 1 (7)* |

** One additional article was identified and is counted together with the seven articles already selected from APA PsycInfo 1 (total of 8 unique articles from APA PsycInfo).*

Note: * (asterisk) denotes truncation of search terms to broaden retrieval. Boolean operator OR was used within search blocks; AND was used to combine search blocks. A total of 12 unique articles (4 from CINAHL, 8 from APA PsycInfo) were included in the subsequent quality appraisal (see Figure 1, PRISMA flow chart).
